# Editorial for the Special Issue on Inertial Microfluidics

**DOI:** 10.3390/mi12060587

**Published:** 2021-05-21

**Authors:** Soojung Claire Hur, Wonhee Lee

**Affiliations:** 1 Department of Mechanical Engineering and Oncology, Johns Hopkins University, 3400 N Charles St., Latrobe 221, Baltimore, MD 21211, USA; 2 Graduate School of Nanoscience and Technology, Korea Advanced Institute of Science and Technology, 291 Daehak-ro E6-6, Yuseong-gu, Daejeon 34141, Korea; 3 Department of Physics, Korea Advanced Institute of Science and Technology, 291 Daehak-ro E6-6,Yuseong-gu, Daejeon 34141, Korea

The growing demands for label-free, high throughput processing of biological, environmental, and industrial samples have instigated technical innovations for inflow particle manipulations with better resolution and purity. Inertial microfluidics has been providing simple technical solutions to such demands by solely utilizing hydrodynamic forces that have differentially arisen based on flow speed, channel geometry, and particle properties. In this Special Issue of *Micromachines*, we showcase reviews and original research manuscripts reporting recent advancements in the field of Inertial Microfluidics.

About half of the articles in this Special Issue present theoretical and experimental investigations on the fundamentals of inertial microfluidics [1,2,3,4]. Gao et al. [1] report a comprehensive study on inertial focusing and ordering in a square channel. Variation of Reynolds number, Re, from 5 to 280 in a square channel smaller than 100 µm reveals an optimal Re for focusing, and the inertial ordering is found from corner focusing positions. Shen et al. [2] report the result of numerical simulations on low-aspect-ratio semicircular channels with obstacles, which results in the formation of vortices. They show that the magnitude and distributions of Dean vortices and helical vortices can be regulated by the number of obstacles and flow rate, which can help us to understand geometry-induced secondary flow formation mechanism, thereby leading to useful applications for manipulation of microfluidic flows. Choi et al. [3] present the study of inertial focusing of droplets and cells in triangular channels. The focusing positions of deformable viscous droplets are investigated alongside varying Re, deformability, and droplet size. The threshold size of the top-focusing position splitting and the overall path of the focusing position shift are found to be strongly dependent on the size and deformability of droplets. The feasibility of deformability-based cell separation is also shown with MCF10a and MCF7. Rühle et al. [4] present an optimal control method for trajectories of particles in the inertial microfluidic system. An axial control force is applied to control the Saffman effect, which can steer the particles towards the desired position at the outlet. They show that a pulse of particles spread along the channel axis can be steered to a target, and that particles of different radii can be separated most efficiently.

The remainder of this Special Issue includes original research articles on technical innovations of inertial microfluidics for various applications [5,6,7,8]. Kim et al. [5] report the separation of two freshwater, commercially important, microalgal species, namely, Chlorella Vulgaris and Hematococcus Pluvialis. By utilizing a Contraction–Expansion Array (CEA), they purified each species with high purity (>95%) and preserved culturability. This highly pure cell purification enabled the expansion of the target organism without the risk of contamination as the minority organism’s culturability of significantly suppressed. In the article by Du et al. [6], on-chip integration of magnetophoresis and inertial focusing was demonstrated for size-based separation of particles with very small size differences (3, 4, and 5 μm). Through systematic numerical and experimental investigations, the optimum conditions to achieve the preferred particle distribution band separation sufficient for separation were determined to be 0.7 × 10^5^ A/m, 4, and 0.5 for magnetic intensity, sheath flow ratio, and the contraction–expansion ratio, respectively, while maintaining the sum of particle solution and sheath flow flowrate identical. To further reduce the device footprint without sacrificing separation performance, Erdem et al. [7] constructed an elliptic arrangement of spiral microchannels with various aspect ratios and orientations. The elliptical configuration allowed for alternating magnitudes of the Dean drag forces due to alternating radius of curvature; hence, faster separation performance compared to the spinal microchannels arranged in a circular configuration. It was possible to achieve >90% of particle purity from the mixture of 10 and 20 μm microparticles, with the overall device footprint at least 12% less than that of circular spinal channels. Notably, the horizontal elliptic arrangement of the spinal channel outperformed its vertically arranged counterpart when the identical channel geometry and ellipse aspect ratio were implemented. The report by Al-Halhouli et al. [8] describes the fabrication method and particle concentration performance of the trapezoidal microchambers on a spinning compact disk made out of an inert glass wafer. A femtosecond laser machining was tested as a simpler, alternative fabrication method to soft lithography. A five-fold increase in particle concentration was achieved using a very small volume (50 μL) of highly diluted solution (0.0001%). The reported spinning disk particle concentration opens up a new opportunity to concentrate a very small number of particles suspended in a solution with limited volume.

Our Special Issue also published two review papers [9,10]. We hope that the readers find the reviews to be useful for understanding the up-to-date theories of physics of inertial microfluidics [9] and gaining insight into the translational impact that the inertial microfluidics has had [10]. Collectively, this Special Issue reports recent advancements in understanding and technological innovations of inertial microfluidics to broaden their applications to real-world and complex samples for high throughput, label-free microscale particle manipulations.
